# Characterization of *TLR1* and expression profiling of TLR signaling pathway related genes in response to *Aeromonas hydrophila* challenge in hybrid yellow catfish (*Pelteobagrus fulvidraco ♀ × P. vachelli ♂*)

**DOI:** 10.3389/fimmu.2023.1163781

**Published:** 2023-03-28

**Authors:** Shengtao Guo, Wenxue Gao, Mengsha Zeng, Fenglin Liu, Qingzhuoma Yang, Lei Chen, Zesong Wang, Yanjun Jin, Peng Xiang, Hanxi Chen, Zhengyong Wen, Qiong Shi, Zhaobin Song

**Affiliations:** ^1^ Key Laboratory of Bio-Resources and Eco-Environment of Ministry of Education, College of Life Sciences, Sichuan University, Chengdu, China; ^2^ Key Laboratory of Sichuan for Fishes Conservation and Utilization in the Upper Reaches of the Yangtze River, College of Life Science, Neijiang Normal University, Neijiang, China; ^3^ Shenzhen Key Lab of Marine Genomics, Guangdong Provincial Key Lab of Molecular Breeding in Marine Economic Animals, BGI Academy of Marine Sciences, BGI Marine, BGI, Shenzhen, China

**Keywords:** hybrid yellow catfish, innate immunity, Aeromonas hydrophila, TLR signaling pathway, expression profile

## Abstract

Toll‐like receptor 1 (TLR1) mediates the innate immune response to a variety of microbes through recognizing cell wall components (such as bacterial lipoproteins) in mammals. However, the detailed molecular mechanism of TLR1 involved in pathogen immunity in the representative hybrid yellow catfish (*Pelteobagrus fulvidraco ♀ × P. vachelli ♂*) has not been well studied. In the present study, we identified the *TLR1* gene from the hybrid yellow catfish, and further comparative synteny data from multiple species confirmed that the *TLR1* gene is highly conserved in teleosts. Phylogenetic analysis revealed distinguishable TLR1s in diverse taxa, suggesting consistence in evolution of the TLR1 proteins with various species. Structural prediction indicated that the three-dimensional structures of TLR1 proteins are relatively conserved among different taxa. Positive selection analysis showed that purifying selection dominated the evolutionary process of TLR1s and TLR1-TIR domain in both vertebrates and invertebrates. Expression pattern analysis based on the tissue distribution showed that *TLR1* mainly transcribed in the gonad, gallbladder and kidney, and the mRNA levels of *TLR1* in kidney were remarkably up-regulated after *Aeromonas hydrophila* stimulation, indicating that TLR1 participates in the inflammatory responses to exogenous pathogen infection in hybrid yellow catfish. Homologous sequence alignment and chromosomal location indicated that the TLR signaling pathway is very conserved in the hybrid yellow catfish. The expression patterns of TLR signaling pathway related genes (*TLR1*- *TLR2* - *MyD88* - *FADD* - *Caspase 8*) were consistent after pathogen stimulation, revealing that the TLR signaling pathway is triggered and activated after *A. hydrophila* infection. Our findings will lay a solid foundation for better understanding the immune roles of TLR1 in teleosts, as well as provide basic data for developing strategies to control disease outbreak in hybrid yellow catfish.

## 1 Introduction

Yellow catfish (*Pelteobagrus fulvidraco*) has become an important economic cultured fish species in China and East Asia due to its rapid growth speed, good taste, and high nutritional values (1). However, intensive aquaculture leads to crowding and excessive stress, which usually result in outbreaks of bacterial infections with high mortality of cultured fishes (2). *Aeromonas hydrophila* is a typical rod-shaped gram-negative bacterium with worldwide distribution in aquatic environments (3, 4). Hydrophilic bacteria are well-studied as fish pathogens, which may cause a series of diseases, including active Aeromonas sepsis in carp, tilapia, perch, catfish and salmon (5).

*A. hydrophila* is the pathogenic bacteria of bacterial hemorrhagic sepsis caused by multidrug-resistant (MDR), which has increased exponentially in the past decade and has reached an alarming rate, leading to a major problem in the aquaculture industry of China (6). At the same time, the outbreak of active motile Aeromonad septicemia (MAS) in various fishes caused by *A. hydrophila* has attracted worldwide attentions (7). In previous studies, researchers observed that tilapia and channel catfish infected with *A. hydrophila* would be damaged in the gills, liver, and intestines, leading to histopathological changes in the infected organs (8, 9).

In the infection process, the innate immune system detect exogenous bacteria *via* sensing cell wall-related components, including lipopolysaccharides, lipoproteins, peptidoglycans, and flagellin (10). Each of these molecules is defined as a pathogen-associated molecular pattern (PAMP) and induces an inflammatory response to realize the host defense (11, 12). The first characterized and most widely studied pattern recognition receptor (PRR) in vertebrates and invertebrates is Toll-like receptors (TLRs) (13, 14). TLRs are important parts of PRR, participating in innate immune defense against exogenous pathogen invasion (15). TLRs represent a large superfamily of type I transmembrane glycoproteins, some of which are shared in multiple species, while others are more restricted in their limited distributions (16).

After pathogen recognition, the Toll/Interleukin-1 receptor (TIR) domain of the TLR triggers downstream signal transductions. Previous findings showed that almost all the TLRs activate the common signaling pathway *via* the TIR-containing linker MyD88, which leads to the activation of NF-κB to promote the transcription of cytokine genes for involvement in the inflammation (17). The interleukin-1 receptor (IL-1R)/TLR superfamily was first identified in 1998 as a protein family containing the Toll-IL-1 receptor domain (18). In that time, many orphan receptors were found in the IL-1R branch, and TLRs had not been proven to be the key innate immune receptor for sensing microbial products (19). Various members of the TLR1 family are usually identified in the genomes of birds and mammals, and they seem to be the consequence of successive rounds of tandem gene replication from the ancestral gene (20). Previous studies on TLR1 have mainly focused on mammals, and it has been reported that TLR2 dimerizes with TLR1 to recognize microbial triacyl lipoproteins, and with TLR6 to recognize diacyl lipopeptides present in mycoplasma, lipoteichoic acid in gram-positive bacteria, or zymosan in yeast (21, 22). The interaction of TLR1 and TLR2 enhances activation of NF-κB in response to synthetic lipopeptides (23). In addition, acylated lipoprotein analogs are recognized by TLR1 at the first time (23). TLR1 can also exert its anti-fungal host defense effect through β-defensin 3, which subsequently activates immune cells through TLR1/TLR2 (24). Certain mutations in the TLR1 affect the susceptibility and immune response to exogenous bacterial pathogens (16).

It has been reported that proteins in living cells interact specifically or non-specifically with a large number of biomolecules (25). To understand the behavior of proteins under macromolecular crowding conditions in cells, it is essential to observe their spatial structure at the atomic level in the physiological environment (26). Areas with essential functions tend to evolve more slowly, and a comparative sequence analysis would be able to identify and characterize the regulatory regions of the genome that have functioned well (27, 28). The evolution rate dN/dS, representing the ratio of non-synonymous substitution rate to synonymous substitution rate, is calculated from the selection pressure of protein coding genes for helping to uncover the mechanism of molecular evolution (29). These above-mentioned methods were employed in our present research in accordance with our previous reports (30, 31).

*TLR1* gene has been cloned and characterized in various fish species, such as Chinese perch (*Siniperca chuatsi*), orange-spotted grouper (*Epinephelus coioides*), Dabry’s sturgeon (*Acipenser dabryanus*) and Atlantic salmon (*Salmo salar*) (32–35). However, the specific molecular mechanism of TLR1 involved in pathogen immune responses remains unknown in hybrid yellow catfish. The published genome at the chromosome-level of the yellow catfish (36) provides a good reference for our present research. In this study, we attempt to characterize the *TLR1* gene (including prediction of protein structures, construction of a phylogenetic tree, and performance of natural selection analysis) for quantification of tissue distribution and its response to bacterial pathogen stimulation, which are helpful for revealing the molecular mechanism of TLR1 in the immune response in hybrid yellow catfish.

## 2 Materials and methods

### 2.1 Identification of *TLR1* from yellow catfish genome

The genome data of yellow catfish were downloaded from NCBI (GenBank assembly accession number: PRJNA494039), and the BLAST search was performed based on protein sequence similarity to identify the *TLR1* gene. As previously reported (36), the TLR1 protein was annotated. The detailed sequence was available for characterization.

### 2.2 Sequence processing and gene synteny analysis

Genomes and corresponding annotation files of *TLR1* in nine representative species, including banded blenny (*Salarias fasciatus*), Crimson tide cichlid (*Pundamilia nyererei*), yellow perch (*Perca flavescens*), Japanese medaka (*Oryzias latipes*), black rockcod (*Notothenia coriiceps*), yellow catfish (*P. fulvidraco*), Tibetan plateau loach (*Triplophysa tibetana*), and bicolor damselfish (*Stegastes partitus*) were downloaded from the NCBI databases. Amino acid sequences of representative TLR1 in different fish species were identified by annotation and confirmed with conserved functional motifs and domains. A comparative genomic survey was conducted to recognize the genetic loci of TLR1 genes in yellow catfish as well as other examined fishes.

### 2.3 Phylogenetic analysis and functional domain identification

To explore the origin and evolution of vertebrate *TLR1* genes, we downloaded TLR1 protein sequences of 71 representative species from the NCBI database (**Supplementary Table 1**). A phylogenetic tree of these TLR1 genes was constructed using the Maximum likelihood method, and the final topology was evaluated with the Poisson correction method (37). Gaps were removed from the entire alignment. Bootstrap values were calculated for evaluation of the stability and reliability of the tree with 1,000 duplications (38). Domain architectures of these TLR1 proteins were predicted using the Simple Modular Architecture Research Tool (39, 40).

### 2.4 Predicted spatial structures of the TLR1 proteins

In this study, the latest version of DeepMind AlphaFold2 (https://github.com/deepmind/alphafold, accessed on 11 August 2022) was used to predict the spatial structures of TLR1 proteins in multiple species, and the corresponding data were visualized using PyMol 2.5 (41, 42).

### 2.5 Modes and strength of natural selection on *TLR1* genes in invertebrates and vertebrates

Synonymous substitutions in protein coding sequences do not result in amino acid variation, which occurs in the third or sometimes the first base of the examined codon (43). Therefore, the gene-level method based on the ratio of non-synonymous (Ka) and synonymous (Ks) substitution rates was used here to detect the potential positive selection signals that are closely related to protein-coding genes in various species (44). The Ka/Ks ratio of 1, <1 or >1 in the coding sequence of the studied protein can be interpreted as neutral mutation, negative (purified) selection, or positive (diversified) selection, respectively. The genomic data of five vertebrate classes (including Mammalia, Amphibia, Aves, Reptilia, and Osteichthyes; 103 species in total) and two invertebrate classes (Insecta and Bivalvia; 13 species in total) from NCBI were examined to determine the origin history of vertebrate-like TLR1s.

To detect the TIR domain architectures of all Toll-like candidates, HMMER was used to blast against the Pfam database (45). Full-length TLR proteins and the TIR domains of each Toll-like candidate were collected for subsequent analyses. For the evolutionary landscape of each TLR gene, the coding sequence alignments were generated by MUSCLE (align codons) (46). Meanwhile, the codeml method implemented in the PAML v4.7 package (47) was employed in this study to estimate dN/dS, dN, number of nonsynonymous substitutions per nonsynonymous site, dS, and number of synonymous substitutions were calculated using Datamonkey webserver (http://classic.datamonkey.org.php, accessed on 11 August 2022) (48, 49).

### 2.6 Identification of chromosome localization of genes involved in the TLR signaling pathway

Genes involved in the TLR signaling pathway were identified by using the KEGG (https://www.genome.jp/kegg/) database. Subsequently, the genome (GCA_022655615.1) of yellow catfish were downloaded and used as the reference genome. Then, *TLR1*, *TLR2*, *Caspase 8*, *MyD88*, *FADD*, *TOLLIP* isoform 1 and *TOLLIP* isoform 2 genes were aligned to the reference genome through tblastn to determine the corresponding position and copy number on the chromosome. Furthermore, the mg2c (version 2.0) online mapping website (http://mg2c.iask.in/mg2c_v2.0/, accessed on 11 August 2022) were employed to visualize the location information of the *TLR1*, *TLR2*, *Caspase 8*, *MyD88*, *FADD*, *TOLLIP* isoform 1 and *TOLLIP* isoform 2 gene.

### 2.7 Sample processing and pathogen challenge

The original group of hybrid yellow catfish were provided by a local fish farm in the suburb of Chengdu City, Sichuan Province, China. We randomly selected 6 fishes from thirty fishes (100 ± 2.0g) for studying the tissue distribution of TLR1 mRNA. Another thirty fishes (100 ± 2.0g) were selected randomly for studying the effect of bacterial infection on TLR1 expression levels. In order to carry out the bacterial infection experiment, we purchased *A. hydrophila* from Shanghai Luwei Technology Co. Ltd. (Shanghai, China). Before the practical experiment, fishes were temporarily fed indoors in a circulating water tank at 28°C for 7 days with commercial feed (protein content at about 40%; Lianyungang Tongwei Feed Co. Ltd., Jiangsu, China). The strain of *A. hydrophila* was inoculated on Luria-Bertani (LB) solid medium and incubated at 37°C for 24 h. A single clone was picked for inoculation in 3 mL LB liquid medium. After incubation for 24 h on a shaker, 1 × 10^6^ CFU/mL bacteria were placed into a turbidimeter tube for turbidity measurement. Number of bacteria was measured using the routine plate counting method.

Six fishes were randomly selected and intraperitoneally injected with 50 μL of phosphate buffered saline (PBS, pH 7.2) as the control group. Another group of 24 fishes were intraperitoneally injected with 50 μL of formalin-treated hydrophile with a concentration of 1.5×10^7^ CFU/Ml in PBS (as the experimental group). Six fishes were randomly selected from the experimental group 24 h after the injection (hpi). The sampled fishes were anesthetized with 300 mg/L MS-222 (Sigma-Aldrich, St. Louis, MO, USA), and then the kidney, skin, liver and gills were collected for subsequent experiments. All animal experiments in this study were approved by the Ethics Committee of the College of Life Sciences, Sichuan University (Permit No. SCU221208001)​.

### 2.8 RNA extraction and reverse transcription PCR (RT-PCR)

According to the manufacturer’s protocol, TRIZOL reagent (Invitrogen, Carlsbad, CA, USA) was used to extract total RNA from various tissues at different stages after the bacterial infection. The RNA samples were processed with DNase to remove potential genomic DNA contamination. Subsequently, agarose gel electrophoresis was performed and a NanoDrop 2000 spectrophotometer (Thermo Scientific, Waltham, MA, USA) was used to evaluate the quality of the separated RNAs. Reverse transcription of RNA samples was conducted using Quantscript Reverse Transcriptase Kit (Tiangen Biotech, Beijing, China). The obtained cDNA solution was used as the template for PCRs.

### 2.9 Quantitative real-time PCR (qRT-PCR)

A CFX Connect Real-Time PCR Detection system (Bio-Rad, Hercules, CA, USA) was used for the qRT-PCR in this study. Three fishes were used for qRT-PCR analysis. The primer sequence for *TLR1*, *TLR2*, *Caspase 8*, *MyD88*, *FADD*, *TOLLIP* isoform 1 and *TOLLIP* isoform 2 were listed in **Table 1**. *β-actin* was selected and used as reference gene and its amplification effect was realized with specific primers (see **Table 1** for sequences). In the PCRs, SuperReal PreMix Plus (SYBR Green) (Tiangen Biotech) was used according to the manufacturer’s protocol. The PCR conducted using the following system, SYBR Premix Ex Taq (from Tiangen Biotech) was 10 μL, the cDNA used here was 40 ng, the final concentration of the primers (referred to anti and sense primer) was 0.2 μM and the making up the final volume of the system to 20 μL with double distilled water. Then the PCR were performed on a Bio-Rad T100 Thermal Cycler amplifier (Bio-Rad, Hercules, CA, USA). with the procedure, initiated 1 min at 95°C, then initiated 5 s at 95°C, annealed 20 s at 60°C and extended 20 s at 72°C. The PCR ends after 40 cycles in the last three steps. At the end of each PCR run, the melting curve analysis was performed in the range of 55°C to 99°C. For each sample, quantitative RT-PCR was performed in triplicate.

**Table 1:** List of the primers used for the fluorescent PCR quantitation.

Primer name	Primer sequence (5’-3’)	Amplicon(bp)
*β-actin F*	GGACCAATCAGACGAAGCGA	105
*β-actin R*	TCAGAGTGGCAGCTTAACCG	
*Toll-1 F*	AACCTTTCTGCTGTCCCCAC	106
*Toll-1 R*	TGGGCGTTCCATGAAAGTCA	
*TLR 2 F*	CGTTTCTGCAAACTCCGCAA	132
*TLR 2 R*	AGGTGTGCGTCTCTAGTCCT	
*Caspase 8 F*	TTGACTCGGTCCGAAAGGTT	70
*Caspase 8 R*	GACTCGGTATTCGTGCTCCA	
*MyD88 F*	ATACGTCCCGTTCCCAAACC	108
*MyD88 R*	GCCGCTGGATGCTTGAATTT	
*FADD F*	AACATCCTCGCAACCTGGAG	127
*FADD R*	GCCGTGTAGTTCAGGTCACA	
*TOLLIP isform x1 F*	TCCGCTCTGTACCCGTAAAC	135
*TOLLIP isform x1 R*	ACCTGTCCTCTCTGAGTGCT	
*TOLLIP isform x2 F*	GCACTTCTGTACAACACGCC	76
*TOLLIP isform x2 R*	CGCAGGCATCTGATACTCCT	

### 2.10 Statistical analysis

Statistical analysis was performed using the popular GraphPad Prim 9 software (GraphPad Software, San Diego, CA, USA) and corresponding calculation was conducted using the routine 2 ^-ΔΔCT^ method (50). All data are represented as mean ± SEM (n=3), and the normality and homogeneity of the variance were checked before statistical analysis. A one-way analysis of variance (ANOVA) was performed to compare the differences in expression levels between different samples.

## 3 Results

### 3.1 Protein sequence and multiple sequence alignment

**Figure 1:**
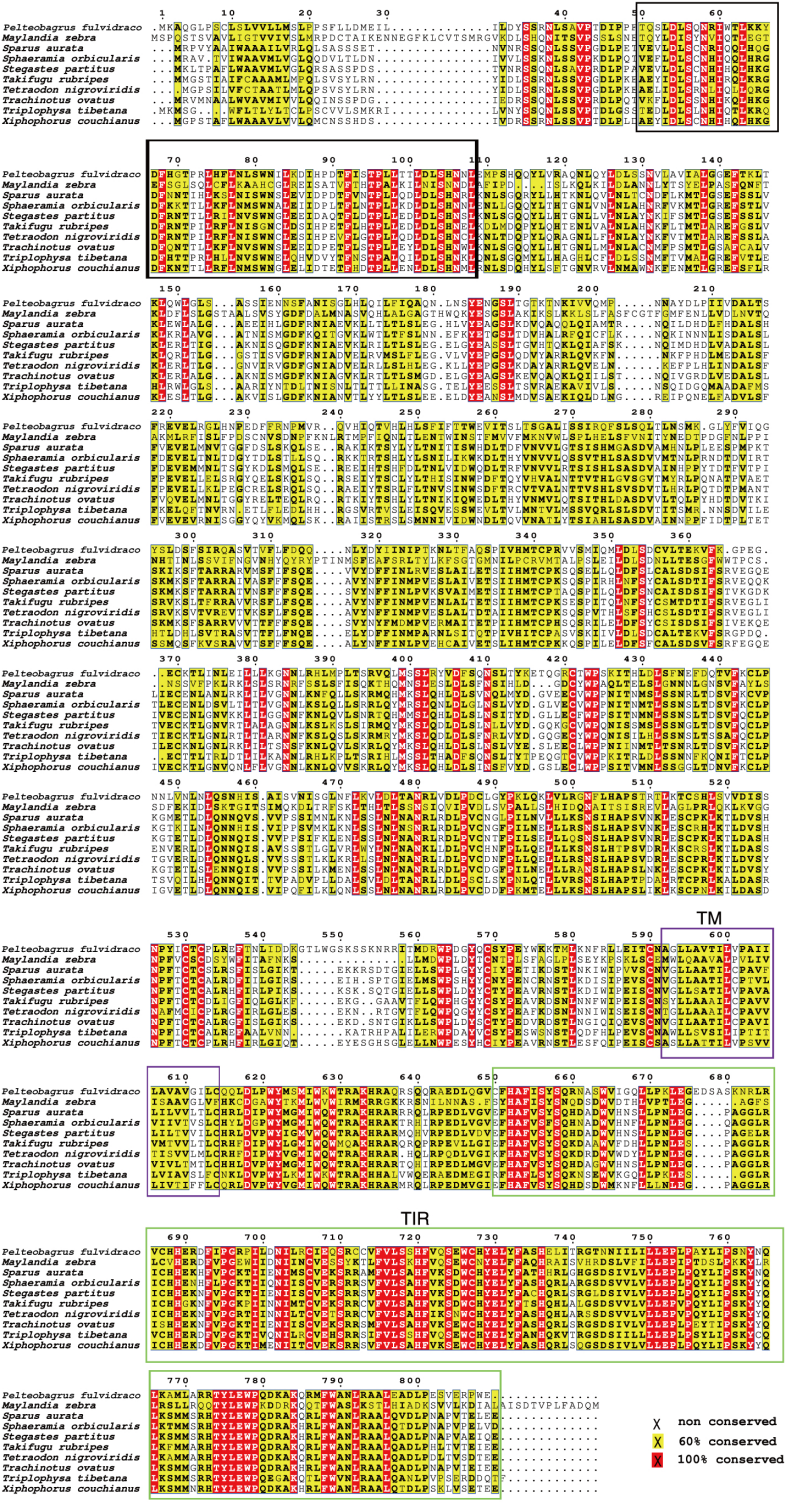
Multiple protein sequences alignment of the TLR1 proteins in nine bony fishes. The black box represents a leucine-rich repeat (LRR) domain, the solid purple box represents the transmembrane (TM) domain, and the green box represents the TIR domain.

Our sequence analysis results show that cDNA of the 9 *TLR1*s range from 2,385 to 3,557 bp, among which 2,385-2,514 bp open reading frames were predicted to encode proteins with 794-837 amino acids (**Table 2**). Multiple protein sequences alignment of TLR1 from ten fishes (including yellow catfish) are summarized in **Figure 1**. Our results showed that a transmembrane domain and the TIR domain were conserved in various fishes, and there is a leucine-rich repeat (LRR) domain in the C-terminus of TLR1s (see more details in **Figure 1**). Our analysis of amino acid sequence homology in different species indicated that TLR1 in yellow catfish exhibited somewhat higher levels of sequence similarity compared to most of the other species TLR1s (**Table 3**). According to similarity analyses in this study, TLR1 in yellow catfish is closely related to TLR1 in striped catfish (*Pangasianodon hypophthalmus*) with similarity of 80.83% (**Table 3**).

**Table 2:** Detailed sequence descriptions of the 9 TLR1 genes.

Species	Nucleotide Accession Number	Full length (bp)	ORF (bp)	5’-UTR (bp)	3’-UTR (bp)	Protein Accession Number	Predicted protein (aa)	Molecular Weight (kDa)	Theoretical pI	Signal peptide	Transmembrane
*Pelteobagrus fulvidraco*	XM_027171867.1	3389	2514	77	789	XP_027027668.1	837	95.69	8.73	NO	YES
*Maylandia zebra*	XM_004553741	2837	2484	31	322	XP_004553798.1	827	93.10	6.85	NO	YES
*Sparus aurata*	XM_030396315	3303	2409	238	656	XP_030252175.1	802	90.89	6.69	YES	YES
*Sphaeramia orbicularis*	XM_030145606	3557	2403	1128	26	XP_030001466.1	800	91.04	8.76	YES	YES
*Stegastes partitus*	XM_008293125	2975	2406	253	316	XP_008291347.1	801	90.45	6.32	YES	YES
*Takifugu rubripes*	XM_003970363	2835	2397	200	238	XP_003970412.2	798	90.73	6.81	YES	YES
*Tetraodon nigroviridis*	EF095150	2587	2391	122	74	ABO15772.1	796	90.69	8.43	NO	YES
*Trachinotus ovatus*	MG762971	3444	2406	610	428	AYM26735.1	801	91.05	6.44	YES	YES
*Triplophysa tibetana*	E1301_Tti023465	2385	2385	0	0	KAA0709838.1	794	90.20	6.57	YES	YES

**Table 3:** Amino Acid similarity of yellow catfish TLR1 to other species TLR1s: To determine the percentage similarity, the TLR1 sequence of yellow catfish was aligned with other species orthologues using CLUSTAL W multiple alignment.

Species	Protein Accession Number	Percentage Similarity (%)
*Pangasianodon hypophthalmus*	XP_026772609.2	80.83%
*Ictalurus punctatus*	XP_017312268.1	79.05%
*Silurus meridionalis*	XP_046722017.1	71.43%
*Colossoma macropomum*	XP_036445680.1	67.45%
*Pygocentrus nattereri*	XP_017565230.1	65.97%
*Myxocyprinus asiaticus*	XP_051505321.1	57.88%
*Triplophysa rosa*	KAI7790121.1	54.94%
*Triplophysa tibetana*	KAA0709838.1	53.22%
*Gymnocypris przewalskii*	ANQ46688.1	54.28%
*Stegastes partitus*	XP_008291347.1	41.28%
*Takifugu rubripes*	XP_003970412.2	39.67%
*Tetraodon nigroviridis*	ABO15772.1	39.43%
*Trachinotus ovatus*	AYM26735.1	41.71%

### 3.2 Comparative synteny and spatial structures of the TLR1 proteins

**Figure 2:**
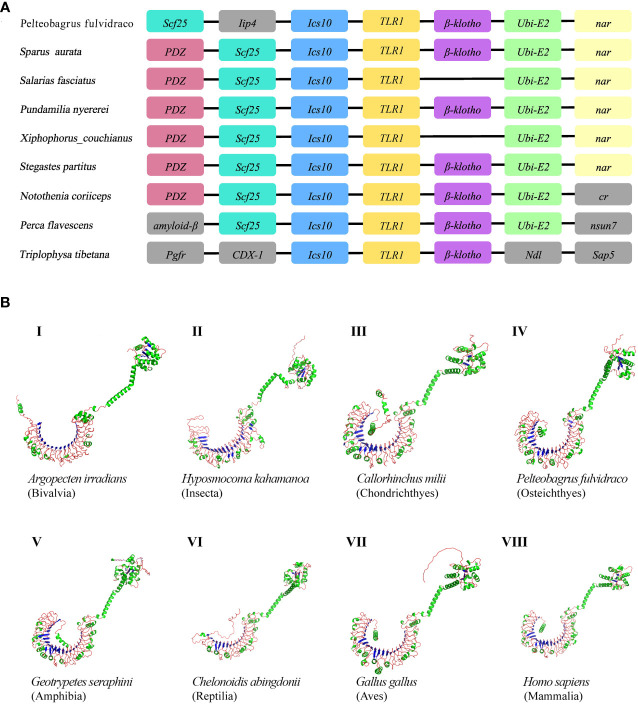
**(A)** Comparative synteny of *TLR1* genes in nine bony fish genomes. Genes and intergenic regions are highlighted with colorful blocks and solid lines, respectively. **(B)** Predicted spatial structures of TLR1 proteins from eight animal taxa. These taxa include Mammalia, Amphibia, Aves, Reptilia, Chondrichthyes, Osteichthyes, Insecta, and Bivalvia. One representative species of each taxon was selected for detailed prediction.

The genetic synteny analysis showed that five genes (including *Scf25*, *Ics10*, *TLR1*, *β-klotho*, and *Ubi-E2*), in great majority of bony fish showed that they formed clusters (**Figure 2A**), although chromosomal rearrangements occurred approximately 465 million years ago (51) due to the differentiation of vertebrates (52, 53). Previous findings revealed that β-Klotho (encoded by Klb) is an obligate co-receptor that mediates FGF21 and FGF15/19 signaling for regulation of glucose and lipid metabolism (54). Bindings of ubiquitin to the substrate is generally considered to occur through the formation of iso-peptide bonds between the C-terminal glycine residue of ubiquitin and the lysine residue of the substrate (55). Individual member of the conserved family of ubiquitin-conjugating enzymes (E2s) mediates the ubiquitination and turnover of the specific substrates in the ubiquitin-dependent degradation pathway (56, 57). However, the current understanding of Scf25 and Ics10 in bony fishes is limited.

AlphaFold is a protein structure prediction tool based on deep learning. It has achieved successes in its highly accurate structure predictions (58). Previous studies showed that the AlphaFold architecture performed high-precision training using only supervised learning of PDB data (59). Multiple sequence alignment (MSA) was added to AlphaFold2 to integrate protein structure and biological information into deep learning algorithms. Our prediction results of the TLR1 spatial structures from eight species are provided in **Figure 2B**. It seems that these spatial structures of TLR1 proteins were mostly horseshoe-shaped, and the corresponding TIR domains are also conserved (in green color on the upper right of **Figure 2B**). However, the reptile TLR1was significantly different from others. The 3D structure of TLR1 in Pinta Island tortoise (*Chelonoidis abingdonii*) looks like a more opened while shorter semicircle (**Figure 2B**, VI), although the overall shape of TLRs is similar with the TIR domain on the top of the semicircle (see **Figure 2B**).

### 3.3 Phylogenetic relationship and domains of TLR1s

**Figure 3:**
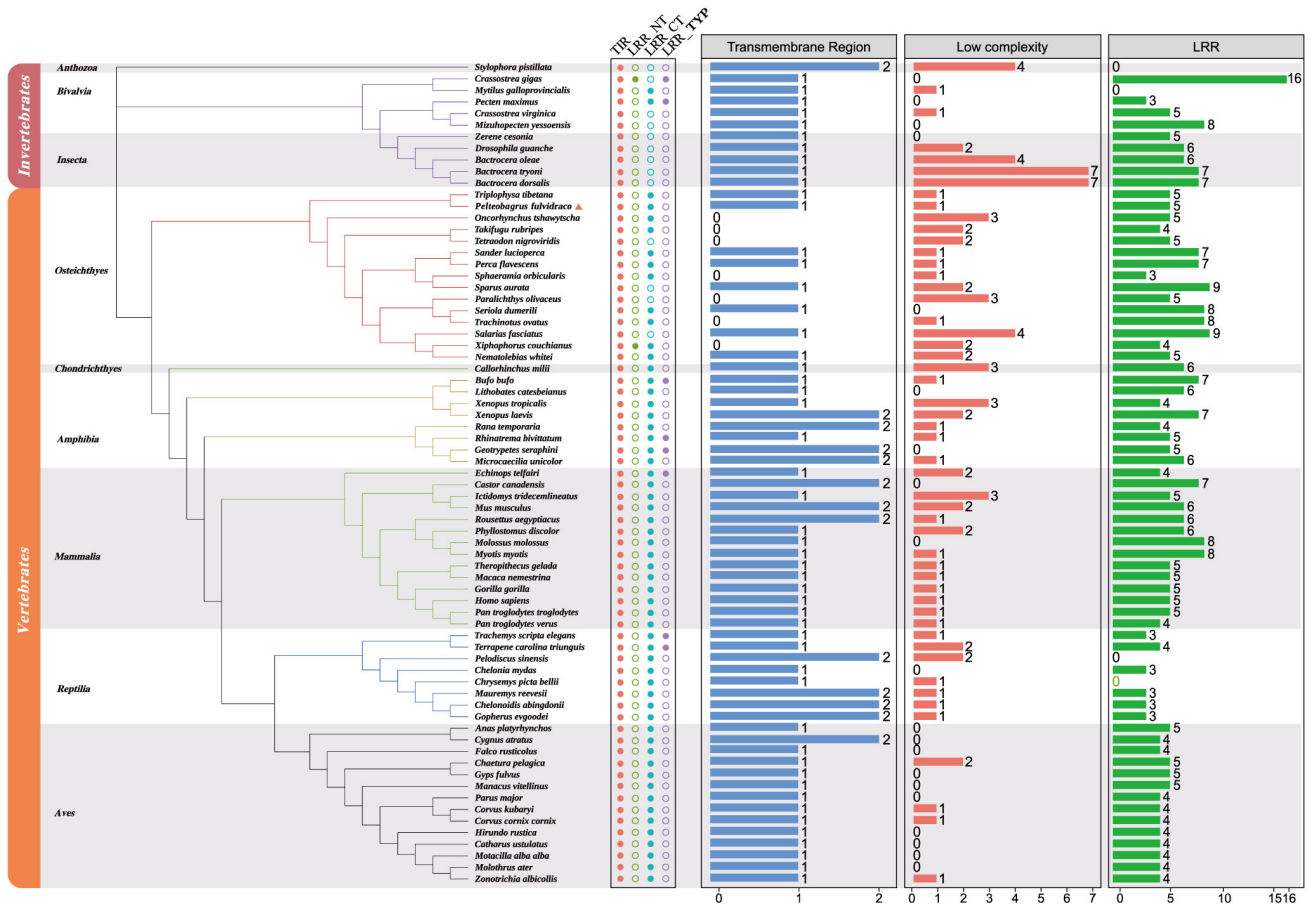
Phylogenetic relationships of TLR1 proteins in various fishes. The phylogenetic tree was constructed with the maximum likelihood method based on a dataset of protein sequences. The stony coral (*Stylophora pistillata*) was used as the outgroup. Genbank accession numbers of these protein sequences are provided in **Supplemental Table 1**. Low complexity, low complexity region; LRR, leucine-rich repeat; LRR_CT, leucine-rich repeat C-terminal; LRR_NT, leucine-rich repeat N-terminal; LRR_TYP, leucine-rich repeats, typical (most populated) subfamily; TIR, Toll/interleukin-1 receptor domain; TR, transmembrane region.

In order to understand the relationship among various TLR1 proteins in both vertebrates and invertebrates, a phylogenetic tree of 71 TLR1 amino acid sequences was constructed using the maximum likelihood method (60, 61). The TLR1 protein sequence from stony coral (*Stylophora pistillata*) was used as the outgroup. According to the well-supported phylogenetic topology (**Figure 3**), our results showed that all the examined TLR1 genes are divided into invertebrate and vertebrate subgroups.

The vertebrate subgroup is further divided into six main branches (including amphibians, mammals, reptiles, birds, bony fishes and cartilaginous fishes), and the invertebrate subgroup is further divided into corals, bivalves and insects. We determined that the topologies generated by the maximum likelihood (ML) (61) and neighbor-joining (NJ) (62, 63) methods (data not shown) are similar, indicating relative stability of the phylogenetic tree. Obviously, the evolution of *TLR1*s is consistent with the evolution of species.

A TLR1 protein typically contains TIR, LRR, LRR_NT, LRR_CT, LRR_TPY, transmembrane domain and low repeat domain (**Figure 3**), but not all TLR1s have the seven domains. However, the TIR domain is present in all the 71 studied species (**Figure 3**), which is the signature signal transduction domain of any TLR and its linker as a scaffold for assembling protein complexes of innate immune signals (64). The TLR1 is missing in some bony fishes (the blue column in **Figure 3**). In fact, this polymorphism of the transmembrane domain was reported to affect the innate immune response to bacterial lipopeptides and the susceptibility to multiple pathogens (65).

### 3.4 Natural selection for TLR1 and TIR domain in both vertebrates and invertebrates and codon-based positive selection

**Figure 4:**
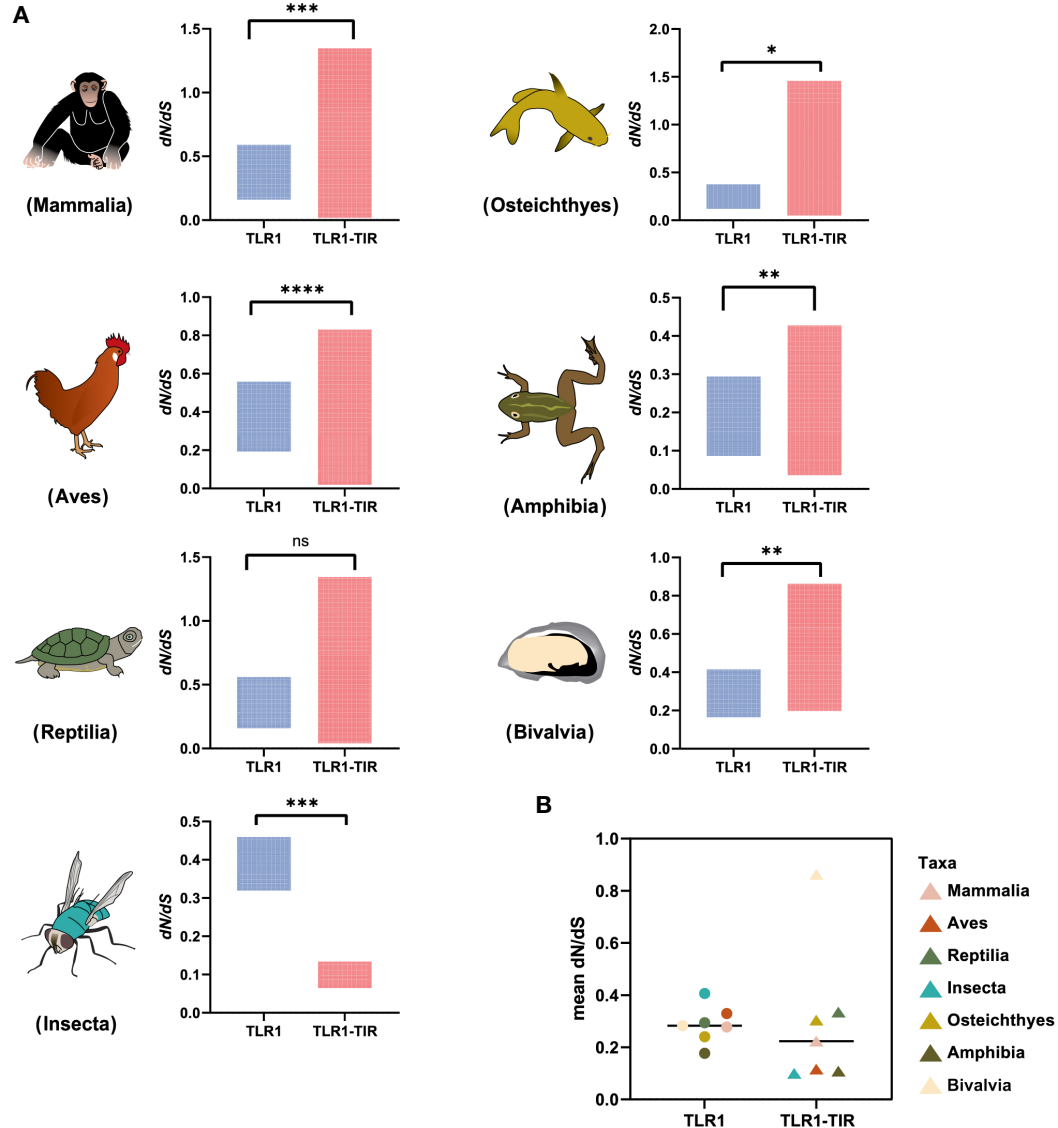
Natural selection analysis of seven animal taxa. **(A)** The dN/dS values of TLR1 and TLR1-TIR domain in representative Mammalia (n=326), Amphibia (n=29), Aves (n=326), Reptilia (n=135), Osteichthyes (n= 301), Insecta (n=16) and Bivalvia (n= 22), “n” is the number of species analyzed for each taxon. **(B)** Comparison of the average dN/dS values of TLR1 and TLR1-TIR domain in the examined taxa. *p<0.05; **p<0.01; ***p<0.001; ****p<0.0001. ns, no significant.

**Figure 5:**
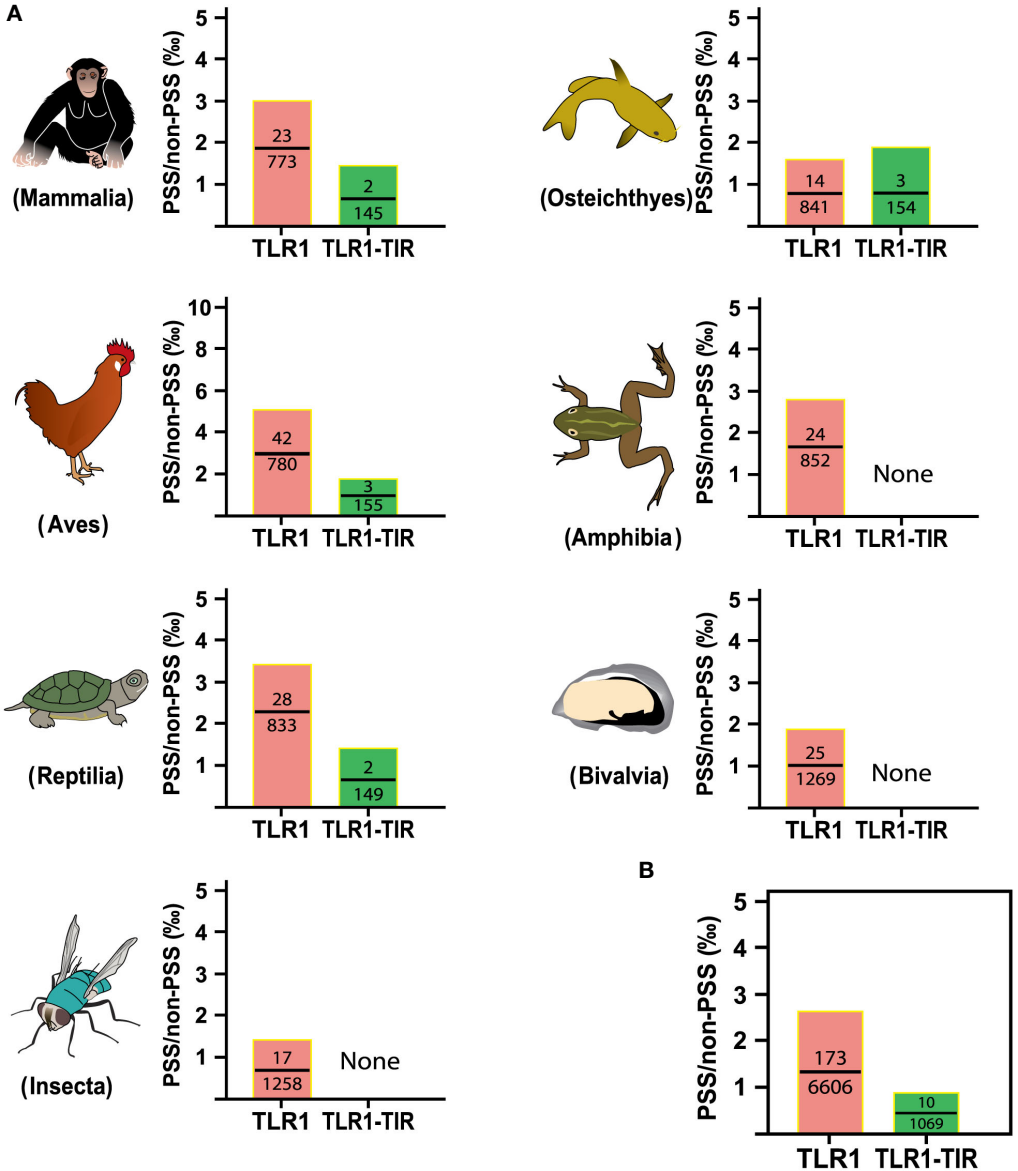
Analysis of positive selection site ratio in seven animal taxa. **(A)** The PSS/non-PSS value of TLR1 and TLR1-TIR domain in representative Mammalia, Amphibia, Aves, Reptilia, Osteichthyes, Insecta and Bivalvia. **(B)** Comparison of average PSS/non-PSS values of TLR1 and TLR1-TIR domain in the examined taxa.

To identify the strength of natural selection of TLR1 in both vertebrates and invertebrates, a selection analysis was performed. The average dN/dS values of TLR1 genes in each taxon were 0.278 (Mammalia), 0.329 (Aves), 0.294 (Reptilia), 0.240 (Osteichthyes), 0.177 (Amphibian), 0.282 (Bivalvia), and 0.406 (Insecta) respectively (**Figure 4A**); the average dN/dS values of TLR1-TIR in each taxon were 0.108 (Amphibia), 0.223 (Mammalia), 0.116 (Aves), 0.335 (Reptilia), 0.100 (Insecta), 0.304 (Osteichthyes), and 0.862 (Bivalvia) (**Figure 4A**) respectively. The dN/dS values of TLR1 and TLR1-TIR in invertebrate lineages are significantly greater than those in vertebrates (**Figures 4A, B**). The results of PSSs/non-PSSs are summarized (see **Figures 5A, B**). It seems that TLR1 genes in the invertebrate lineages exhibited a faster rate of evolution than vertebrates.

### 3.5 Distribution of *TLR1* gene in different tissues

**Figure 6:**
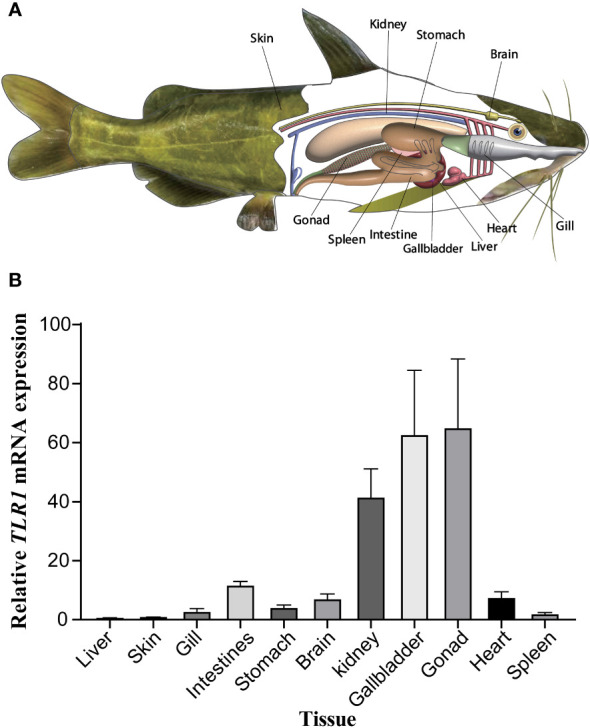
** (A)** Tissue distribution of sampling organs used in this study. The fish model here referred to the hybrid yellow catfish. **(B)** Relative transcription patterns of *TLR1* gene in 11 examined tissues of the hybrid yellow catfish. *β-actin* was selected and used as the reference gene. Each error bar represents a standard error of the mean values (n = 3).

The tissue distribution of sampling organs of hybrid yellow catfish used in this study were exhibited in **Figure 6A**. The tissues-specific expression levels (**Figure 6B**) demonstrated that the *TLR1* gene was widely transcribed in all the 11 tested tissues (including gallbladder, intestine, liver, stomach, heart, gills, spleen, kidney, skin, gonad and brain), but exhibited somehow tissue preference. The *TLR1* gene has high mRNA levels in the gonad, gallbladder and kidney, which are important components of the immune system in fishes.

### 3.6 Responses of *TLR1* gene to exogenous *A. hydrophila* infection

**Figure 7:**
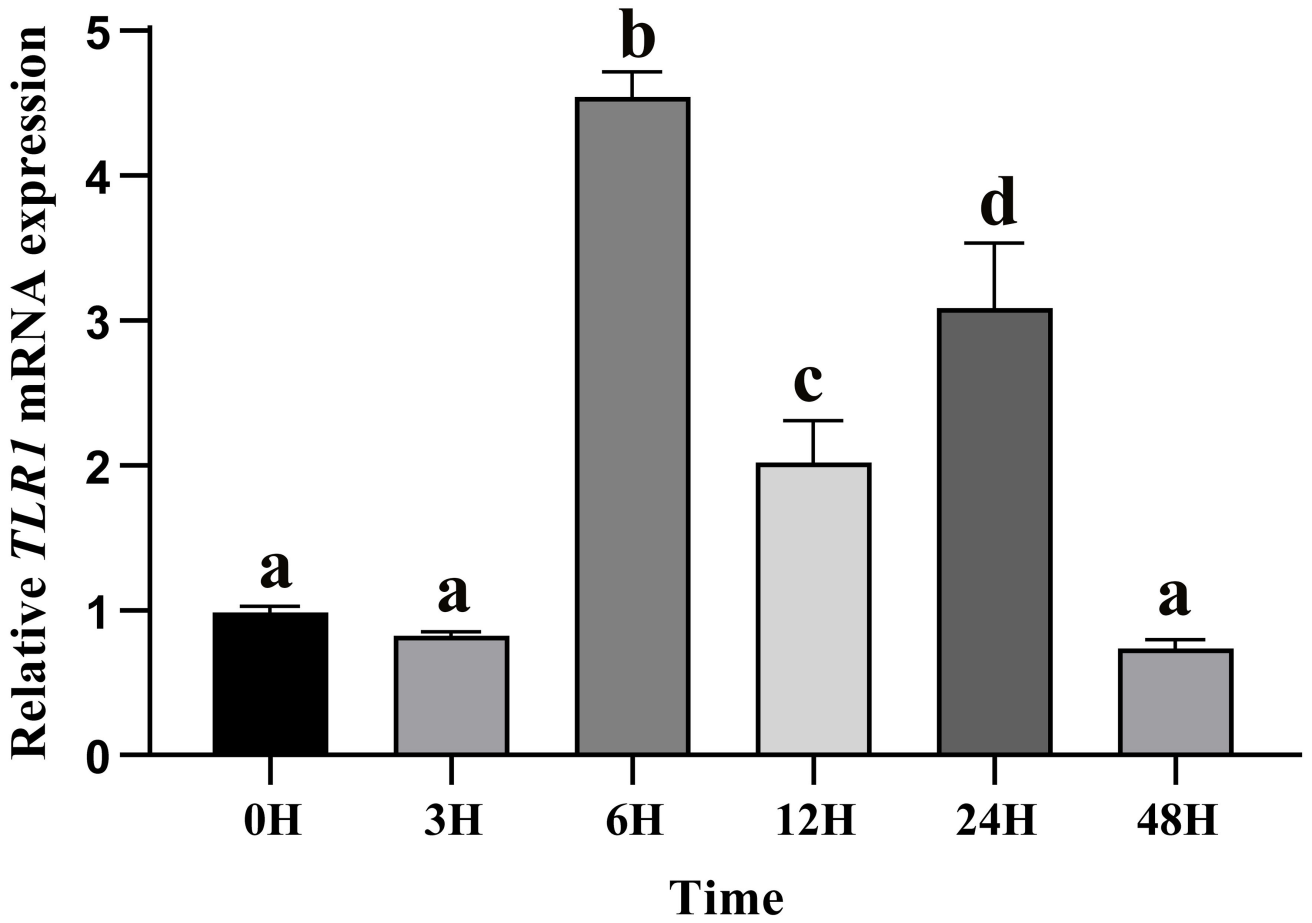
Relative transcription levels of *TLR1* gene in the kidney of hybrid yellow catfish infection of exogenous *A.hydrophila*. *β-actin* was selected and used as the reference gene. Different letters above the bars represent significant differences among the examined groups. Each error bar represents a standard error of the mean values (n = 3).

After *A. hydrophila* infection, expression levels of *TLR1* gene at different time points in hybrid yellow catfish were measured. Our results showed that *TLR1* gene responded to bacterial attack, but the response pattern is tissue-specific (**Figure 7**). *TLR1* mRNA levels in kidney were remarkably up-regulated after the bacterial infection.

### 3.7 Chromosome location of genes involved in the TLR signaling pathway

**Figure 8:**
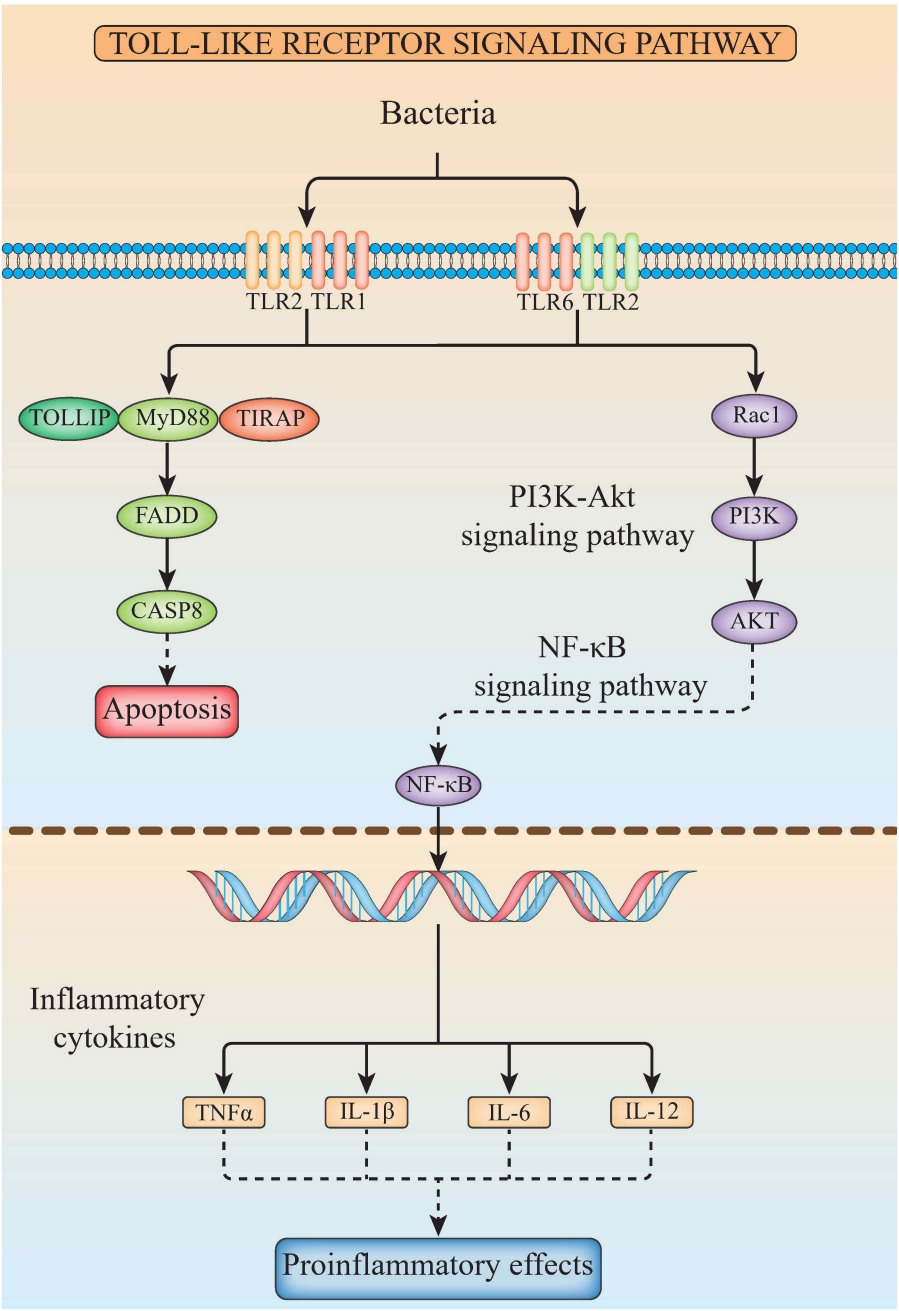
The diagram of TLR signaling pathways in hybrid yellow catfish, from top to bottom are extracellular, cytoplasmic and nuclear.

**Figure 9:**
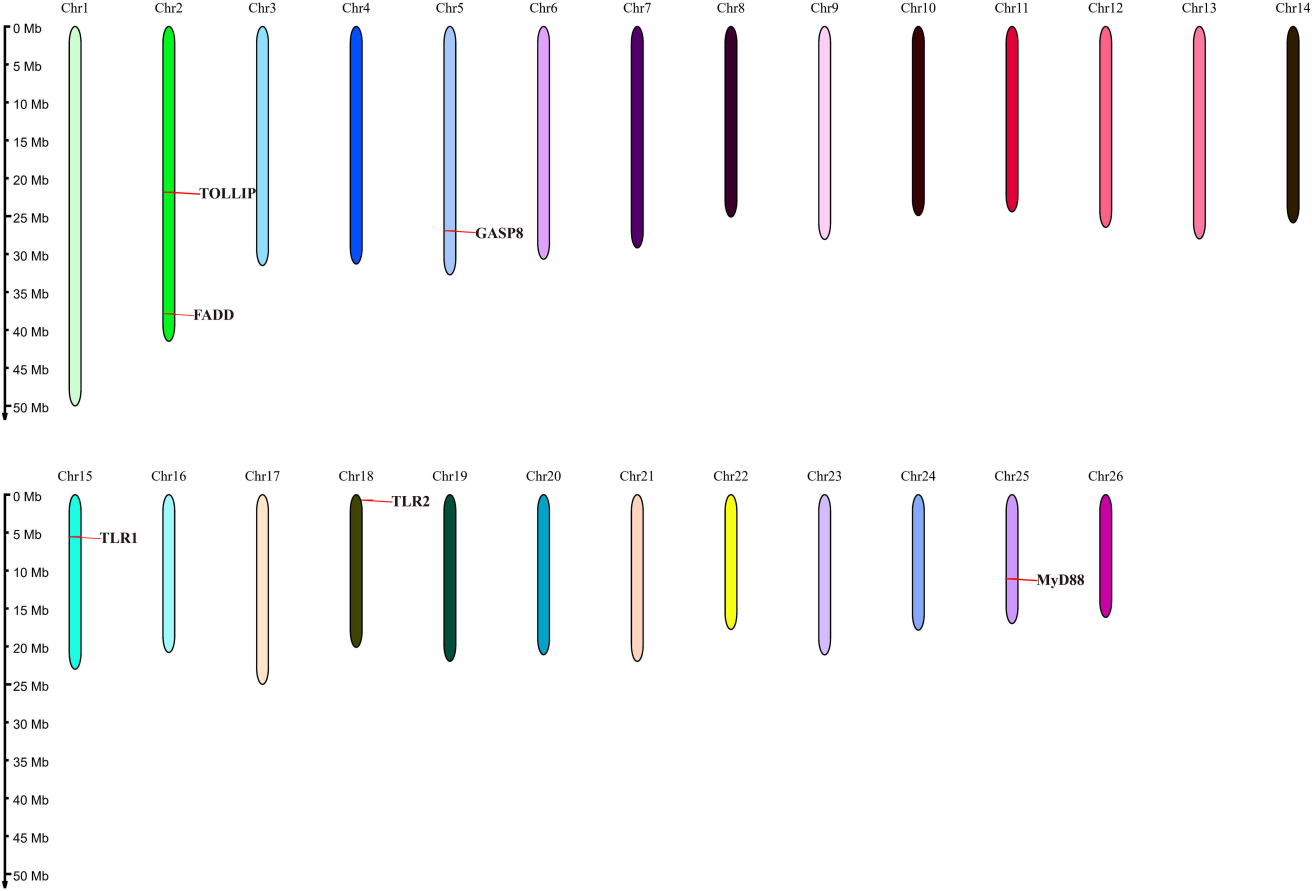
Location of TLR signaling pathway genes on the chromosome of yellow catfish.

The related genes involved in TLR signaling pathway are shown in **Figure 8A**, in which (*TLR1*-*TLR2*) - *MyD88* - *FADD*- *Caspase 8* composed of TLR signaling pathway finally induces apoptosis (**Figure 8**). Our results showed that there are 26 chromosomes in the genome of the yellow catfish, among which the longest chromosome is Chr 1 with the length of 52.4 Mb, and the shortest chromosome is Chr 26 with the length of 16.9 Mb (**Figure 9**). And we found that *TLR1* was located at 5815026-5818519 of chromosome 15 with a single copy in the yellow catfish (GCA_022655615.1) genome (**Figure 9** and **Table 4**). *TOLLIP* and *FADD* located on chromosome 2. *Caspase 8*, *TLR2* and *MyD88* located on chromosome 5, 18 and 25, respectively. Refer to **Table 4** for the exact location of TLR signaling pathway genes on different chromosomes.

**Table 4:** Chromosomal location of TLR signaling pathway genes.

Gene name	Copy number	Chromosome number	Location
*TLR1*	1	Chr15	5815026.5818519
*TLR2*	1	Chr 18	872501.877500
*Caspase 8*	1	Chr 5	27862705.27868325
*MyD88*	1	Chr 25	14613625.14617435
*FADD*	1	Chr 2	38107238.38109371
*TOLLIP isform x1*	1	Chr 2	22261977.22276059
*TOLLIP isform x2*	1	Chr 2	22261977.22276059

### 3.8 Expression profiling of genes involved in the TLR signaling pathway in response to *A. hydrophila* challenge in the in hybrid yellow catfish

**Figure 10:**
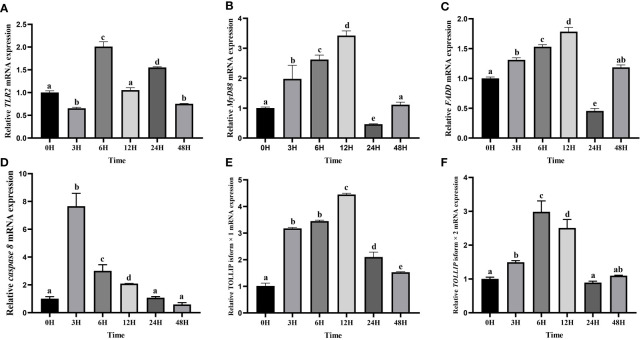
Relative expression levels of TLR signal pathway related genes in kidney after the *A*. *hydrophila* challenge. Transcriptional changes of hybrid yellow catfish *TLR2* **(A)**, *MyD88* **(B)**, *FADD* **(C)**, *caspase 8* **(D)**, *TOLLIP* isoform 1 **(E)** and *TOLLIP* isoform 2 **(F)** in kidney were analyzed at 0, 3, 6, 12, 24 and 48 H after *A*. *hydrophila* challenge.

Then the expression levels of *TLR2*, *MyD88*, *FADD*, *TOLLIP* isoform 1 and *TOLLIP* isoform 2 involved in the TLR signaling pathway were tracked at six time points (0 h, 3 h, 6 h, 12 h, 24 h, 48 h). Our results showed that transcript levels of all these genes were upregulated after the *A. hydrophila* stimulation (**Figure 10**). Among the six time points, the expression pattern of *TLR2* was wavy, and reached the maximum at 6 h (**Figure 10A**). *MyD88* and *FADD* exhibited a similar expression pattern, which gradually increased before the 12 h, reached the maximum at 12 h, and showed a process of first down-regulation and then up-regulation in the subsequent 24 h and 48 h (**Figures 10B, C**). The expression of *Caspase 8* gene reached the maximum at 3 H, and showed a downward trend at the subsequent time points (**Figure 10D**). The expression levels of *TOLLIP* isoform 1 and *TOLLIP* isoform 2, two isoforms of *TOLLIP*, also exhibited a similar expression pattern, which reached the highest values at 12 H and 6 H, respectively (**Figures 10E, F**).

## 4 Discussion

In this study, the hybrid yellow catfish *TLR1* gene was identified for further characterization and investigation. Protein sequence analysis showed that a transmembrane domain and the TIR domain were conserved in various fishes (**Figure 1**). Our comparative synteny data from multiple species confirmed that the identified *TLR1* gene do exist in the yellow catfish genome (**Figure 2A**). Predicted spatial structures of each TLR1 is typical horseshoe shaped with well-positioned pockets (**Figure 2B**) to accommodate the triacylated lipopeptide ligand (59). It seems that the TLR1s from different taxa are relatively conserved, although reptilia present many variances (**Figure 2B**, VI). The phylogenetic tree of TLR1 proteins from 71 representative species is divided into two branches of vertebrates and invertebrates (**Figure 3**). Among these species, there are three classes of invertebrates (Anthozoa, Bivalvia, and Insecta) and six classes of vertebrates (Mammalia, Amphibia, Aves, Reptilia, Chondrichthyes, and Osteichthyes). Our genomics survey showed that the main functional domains of TLR1 proteins are LRR, LRR-C-terminal (LRR_CT) motifs and LRR-N-terminal (LRRNT) motif in the extracellular domain, the transmembrane domain and a TIR in the cytoplasmic domain, low complexity region and LRR_TYP motif (LRR-TYP represents typical LRRs, whereas LRR represents LRR outliers) (**Figure 3**). TLR1 proteins of the analyzed 71 species share the TIR -domain, while most TLR1 proteins in invertebrates lost the LRR_CT (**Figure 3**). Positive selection analysis showed that purifying selection dominated the evolutionary process of TLR1s and TLR1-TIR domain in both vertebrates and invertebrates (**Figure 4**). The results of PSSs/non-PSSs show that TLR1 genes in the invertebrate lineages exhibited a faster rate of evolution than vertebrates (**Figure 5**). qRT-PCR was applied to detect tissue distribution of the *TLR1* transcript in yellow catfish, and our results demonstrated detection in all the 11 selected tissues while high levels in the immune system (**Figure 6**). Chromosomal mapping results showed that *TLR1*, *TLR2*, *caspase 8*, *MyD88*, *FADD*, *TOLLIP isoform 1* and *TOLLIP isoform 2* involved in the TLR signaling pathway were single-copy genes, implying that the genes of the TLR signaling pathway was relatively conserved in the yellow catfish (**Figure 9** and **Table 4**). After infection with *A. hydrophila*, the transcriptional levels of *TLR1*, *TLR2*, *caspase 8*, *MyD88*, *FADD*, *TOLLIP* isoform 1 and *TOLLIP* isoform 2 were all significantly up-regulated in the kidney, indicating that bacterial infection induced the activation of TLR signaling pathway in the yellow catfish (**Figure 10**).

TIR domain is the representative signal domain of TLRs and their adaptors, serving as a scaffold for assembling protein complexes in the innate immune signal transductions (64, 66). Proteins with TIR domain have also been identified in plants, where they mediate disease resistance (67); however, TIR domain containing proteins in bacteria are involved in virulence (61). In this study, TLR1s of all the 71 examined species shared the TIR domain, implying that TIR may be a functional domain necessary for TLR1 to trigger the immune response in yellow catfish. Effect of the loss of LRR_CT in vertebrate TLR1 is unknown, presumably because genes are accompanied by a certain extent in the process of evolution. Interestingly, previous studies reported that TLRs form homodimers and heterodimers within the membrane, and the single transmembrane domain of these receptors has been involved in the dimerization and corresponding functions (68, 69). The loss of transmembrane domain in TLR1 proteins of some bony fishes may inactivate the formation of dimers in the membrane and further affect its function. We speculate that this evolution may be related to the aquatic environment for fishes’ growth. The small variances in protein spatial structure of TLR1s from different taxa indicated that the function of this protein in various species is relatively conserved (70, 71).

In general, the TLR proteins in prototype metazoans are structurally characterized by three typical domains, including hydrophobic tandem LRR extracellular domain (ECD, mediating PAMP recognition), a short transmembrane (TM) domain, and the intracellular TIR (for signal transmission to downstream pathway components) (72). In our present study, the pattern and strength of natural selection that affects the TLR1s and TLR1-TIRs in both vertebrates and invertebrates were well analyzed. The values of dN/dS (ω) indicate that all the animal TLR1 genes are under purification selection (the value ranges from 0.177 in Amphibian to 0.406 in Insecta for TLR1 genes; **Figure 4**). A number of functional residues of TLR1 proteins in different species are highly conserved, although they are located directly at the host-environment interface, providing a rigid structural framework for identifying the conserved pathogen-associated molecular patterns (73). The cytoplasmic TIR signal domain induces a signal cascade when the TLR recognizes a specific ligand (74). With average dN/dS (ω) values of TLR1-TIR ranging from 0.100 for Insecta to 0.862 for Bivalvia, it seems that TLR1-TIR exhibited better evolutionary flexibility than TLR1 (**Figure 4**); that is to say, the differences in the activity levels among different taxa TLR1-TIR domains and downstream molecular components are particularly obvious. The mean values of PSS/non-PSS ranged from 17/1258 in Insecta to 42/780 in Aves (**Figure 5**), suggesting that TLR1 in Aves is potentially more tolerant for non-synonymous mutations, which may be subjected to positive selection and fixation in Aves (75).

Previous studies showed that tissue distribution analysis helps us to understand the relative expression levels of target genes in various tissues (76, 77), and to determine the detailed biological processes with functional genes. Our results in this study revealed widespread presence of *TLR1* transcript in all the 11 collected tissues (**Figure 6**), indicating that *TLR1* is ubiquitous in hybrid yellow catfish. Meanwhile, *TLR1* exhibited a similar transcription pattern in *Acipenser dabryanus* (76). Interestingly, the mRNA levels of *TLR1* in immune system were much higher (**Figure 6**), indicating that TLR1 may mainly function for immunity response in hybrid yellow catfish. Our further data demonstrated that the transcription levels of *TLR1* increased significantly after the infection of exogenous *A. hydrophila* when compared with the control group (**Figure 7**), revealing that TLR1 participated in bacterial infection in hybrid yellow catfish.

In mammals, apoptosis of the infected cells prevented the spread of microbes throughout the whole body, a strategy that has been maintained throughout the evolution. Detection of specific glycopeptides in the cell wall peptidoglycan triggers the activation of caspase-8-dependent apoptosis, thereby enhancing the clearance of bacteria in the infected cells (78). TLR2, in conjunction with TLR1, plays an important role in the innate immune response by recognizing microbial lipoproteins and lipopeptides in the process of infection (21). Apoptosis signals triggered by TLR2 were functions with MyD88, and this pathway involves FAS-associated death domain protein (FADD) and caspase 8. Furthermore, the binding of MyD88 to FADD is sufficient to induce the cell apoptosis (79). However, TLR signaling pathway consisting of (TLR1-2) - MyD88 - FADD - Caspase 8 has not been systematically studied in teleosts. In this study, *TLR1*, *TLR2*, *caspase 8*, *MyD88*, *FADD* and *TOLLIP* were successfully identified in the yellow catfish, and we proved that there is an only single copy of these genes through the homologous sequence alignment and chromosome location, indicating that TLR signaling pathways are very conserved in the yellow catfish. The expression profiling of TLR signaling pathway genes after pathogen stimulation showed that the infection of pathogens induced the activation of (TLR1-2) - MyD88 - FADD - Caspase 8 involved signaling pathway, and inhibits the spread of bacteria in the body through cell apoptosis.

## 5 Conclusions

In summary, the *TLR1* gene of hybrid yellow catfish was characterized using the comparative genomic survey. The phylogenetic analysis of TLR1 revealed the evolutionary relationship of the selected animals. Our study showed that TLR signaling pathway is very conserved and indispensable in bacterial infection in the hybrid yellow catfish. Meanwhile, our research provides a reference dataset for in-depth studies on the molecular mechanisms of (TLR1-2) - MyD88 - FADD - Caspase 8 mediating apoptosis pathway in bony fish after the bacterial pathogen infection.

## Data availability statement

The original contributions presented in the study are included in the article/**Supplementary Material**. Further inquiries can be directed to the corresponding authors.

## Ethics statement

The animal study was reviewed and approved by the Ethics Committee of the College of Life Sciences, Sichuan University.

## Author contributions

SG designed the experiments. SG, WG and MZ performed the experiments. SG analyzed the data. SG, WG, MZ, QY, FL, LC, Z-YW, YJ and PX contributed reagents/materials tools. SG contributed to the discussion. SG wrote the draft manuscript. ZS, QS and Z-YW supervised the project and revised the manuscript. Z-YW acquired the fund. All authors contributed to the article and approved the submitted version.
