# Supplementary material for: Characterization of TLR1 and expression profiling of TLR signaling pathway related genes in response to Aeromonas hydrophila challenge in hybrid yellow catfish (Pelteobagrus fulvidraco ♀ × P. vachelli ♂)
Source: Front Immunol. 2023 Mar 28;14:1163781. doi: 10.3389/fimmu.2023.1163781 (PMC10086376; doi:10.3389/fimmu.2023.1163781)
Supplement: Supplementary file 1 [file Table_1.docx]

**Table S1.** NCBI accession numbers of protein sequences

| Species name | Protein name | Accession number |
| --- | --- | --- |
| ***Stylophora pistillata*** | toll-like receptor 1 | PFX15166.1 |
| ***Crassostrea gigas*** | toll-like receptor 1 | ADV16385.1 |
| ***Mytilus galloprovincialis*** | toll-like receptor 1 | VDI78751.1 |
| ***Pecten maximus*** | toll-like receptor 1 | XP 033755286.1 |
| ***Crassostrea virginica*** | toll-like receptor 1 | XP 022300603.1 |
| ***Mizuhopecten yessoensis*** | toll-like receptor 1 | XP 021346851.1 |
| ***Zerene cesonia*** | toll-like receptor 1 | XP 038219817.1 |
| ***Drosophila guanche*** | toll-like receptor 1 | XP 034133199.1 |
| ***Bactrocera oleae*** | toll-like receptor 1 | XP 036214140.1 |
| ***Bactrocera tryoni*** | toll-like receptor 1 | XP 039966929.1 |
| ***Bactrocera dorsalis*** | toll-like receptor 1 | JAC38197.1 |
| ***Triplophysa tibetana*** | toll-like receptor 1 | KAA0709838.1 |
| ***Pelteobagrus fulvidraco*** | toll-like receptor 1 | XP_027027668.1 |
| ***Oncorhynchus tshawytscha*** | toll-like receptor 1 | XP 024238500.1 |
| ***Takifugu rubripes*** | toll-like receptor 1 | XP 003970412.2 |
| ***Tetraodon nigroviridis*** | toll-like receptor 1 | ABO15772.1 |
| ***Sander lucioperca*** | toll-like receptor 1 | XP 031175177.1 |
| ***Perca flavescens*** | toll-like receptor 1 | XP 028445178.1 |
| ***Sphaeramia orbicularis*** | toll-like receptor 1 | XP 030001466.1 |
| ***Sparus aurata*** | toll-like receptor 1 | XP 030252175.1 |
| ***Paralichthys olivaceus*** | toll-like receptor 1 | AFW04264.1 |
| ***Seriola dumerili*** | toll-like receptor 1 | XP 022607278.1 |
| ***Trachinotus ovatus*** | toll-like receptor 1 | AYM26735.1 |
| ***Salarias fasciatus*** | toll-like receptor 1 | XP 029970757.1 |
| ***Xiphophorus couchianus*** | toll-like receptor 1 | XP 027865028.1 |
| ***Nematolebias whitei*** | toll-like receptor 1 | XP 037531784.1 |
| ***Callorhinchus milii*** | toll-like receptor 1 | XP 007887373.1 |
| ***Bufo bufo*** | toll-like receptor 1 | XP 040274920.1 |
| ***Lithobates catesbeianus*** | toll-like receptor 1 | QYZ87002.1 |
| ***Xenopus tropicalis*** | toll-like receptor 1 | XP 031751229.1 |
| ***Xenopus laevis*** | toll-like receptor 1 | XP 018084600.1 |
| ***Rana temporaria*** | toll-like receptor 1 | XP 040191068.1 |
| ***Rhinatrema bivittatum*** | toll-like receptor 1 | XP 029445281.1 |
| ***Geotrypetes seraphini*** | toll-like receptor 1 | XP 033803378.1 |
| ***Microcaecilia unicolor*** | toll-like receptor 1 | XP 030047152.1 |
| ***Echinops telfairi*** | toll-like receptor 1 | XP 004703408.1 |
| ***Castor canadensis*** | toll-like receptor 1 | JAV44399.1 |
| ***Ictidomys tridecemlineatus*** | toll like receptor 1 | KAG3276766.1 |
| ***Mus musculus*** | toll-like receptor 1 | AAG35062.1 |
| ***Rousettus aegyptiacus*** | toll like receptor 1 | KAF6432594.1 |
| ***Phyllostomus discolor*** | toll like receptor 1 | KAF6133350.1 |
| ***Molossus molossus*** | toll like receptor 1 | KAF6503058.1 |
| ***Myotis myotis*** | toll like receptor 1 | KAF6389189.1 |
| ***Theropithecus gelada*** | toll-like receptor 1 | XP 025242513.1 |
| ***Macaca nemestrina*** | toll-like receptor 1 | XP 024650516.1 |
| ***Gorilla gorilla*** | toll-like receptor 1 | AGR82338.1 |
| ***Homo sapiens*** | toll-like receptor 1 | AAC34137.1 |
| ***Pan troglodytes troglodytes*** | toll-like receptor 1 | AGR82382.1 |
| ***Pan troglodytes verus*** | toll-like receptor 1 | AGR82414.1 |
| ***Trachemys scripta elegans*** | toll-like receptor 1 | XP 034628693.1 |
| ***Terrapene carolina triunguis*** | toll-like receptor 1 | XP 024077328.2 |
| ***Pelodiscus sinensis*** | toll-like receptor 1 | XP 006129587.1 |
| ***Chelonia mydas*** | toll-like receptor 1 | XP 043400456.1 |
| ***Chrysemys picta bellii*** | toll-like receptor 1 | XP 023962466.1 |
| ***Mauremys reevesii*** | toll-like receptor 1 | XP 039395842.1 |
| ***Chelonoidis abingdonii*** | toll-like receptor 1 | XP 032631230.1 |
| ***Gopherus evgoodei*** | toll-like receptor 1 | XP 030420150.1 |
| ***Anas platyrhynchos*** | toll-like receptor 1 | XP 038033957.1 |
| ***Cygnus atratus*** | toll-like receptor 1 | XP 035410088.1 |
| ***Falco rusticolus*** | toll-like receptor 1 | XP 037247147.1 |
| ***Chaetura pelagica*** | toll-like receptor 1 | KFU95976.1 |
| ***Gyps fulvus*** | toll-like receptor 1 | ABF19730.1 |
| ***Manacus vitellinus*** | toll-like receptor 1 | KFW83859.1 |
| ***Parus major*** | toll-like receptor 1 | XP 015480343.1 |
| ***Corvus kubaryi*** | toll-like receptor 1 | XP 041909274.1 |
| ***Corvus cornix cornix*** | toll-like receptor 1 | XP 010400100.3 |
| ***Hirundo rustica*** | toll-like receptor 1 | XP 039920509.1 |
| ***Catharus ustulatus*** | toll-like receptor 1 | XP 032916172.1 |
| ***Motacilla alba alba*** | toll-like receptor 1 | XP 037990632.1 |
| ***Molothrus ater*** | toll-like receptor 1 | XP 036238158.1 |
| ***Zonotrichia albicollis*** | toll-like receptor 1 | XP 005490559.1 |
